# 

**Published:** 1863-10

**Authors:** 


### INDEX TO VOL. III.

Alienation, on a peculiar form of mental, caused by the political and social disturbances in France during February, 1848, by Dr. Bergeret, 608
Albers, Dr., on epilepsy, 752
Armstrong, Sir Wm., on the consumption of coal,
Asylums, American, for the insane, 286
"Aurora Floyd," 585
Bavaria, suicide in, 579
Behr, Dr., on the criminal responsibility of deaf mutes, 183
Bergeret, Dr., on a peculiar form of mental alienation caused by political and social disturbances in France in 1848, 608
Bethlehem hospital, 370, 558
Body, on cooling of, after death. By Dr. B. W. Richardson, 24
British Medical Association, 604
Bouchardat, on work and its influence on health, 321
Browne, Dr. J. Crichton, on mania ephemera, 45
Buchanan's, Dr., report on health of cotton-workers in Lancashire, 261
Butcher's, Mr., surgical reports, 641
Carus on the unconscious life of the soul, 132
Certainty, on the law of, 432
Certificates, medical, in lunacy, 155
Clarke, Mr. J. Lockhart, F.R.S., on volition, psychologically and physiologically considered, 1, 193
olonization of insane, 576
Comparative medicine, 489
Constans, Dr., on an epidemic of hysterical demonomania, 189
Cotton famine, the, 261, 561
Convict discipline,
Council, the medical, 554
Crime, the medical evidence of, 363
Criminals, mental diseases of, by Dr. Moriz, 758
Dangerous classes, 136
Davies, the late Mr. J. W., 768
——— Mr. J. Alexander, on a new theory of vision, 92
——————— on the influence of material objects upon the mind, 234
——————— on some of the operations of the human understanding, 467
——— Rev. G. W., on the law of certainty, 432
Deaf mutes, on the criminal responsibility of, by Dr. Behr, 183
Demonomania, on an epidemic of hysterical, by Dr. Constans, 190
Dublin, the, school of surgery, 641
Du Saulle (Dr. Legrand), on partial responsibility in lunacy and nervous disorders, 668
Etherization, the discoverer and discovery of, 519
Epilepsy, Dr. Albers, on, 725
Federal volunteers, on the sickness and mortality of, 123
Female physicians, 171
Fevers, continued, Dr. Murchison on, 702
France, the insane in, 340
Garibaldi's wound, 171
George III., lunacy of, the *Times* on, 766
Gheel, Dr. Parigot on, 190
Ghosts, patent and prescriptive, 394
Goadby, Mr. Edwin, on phrenology and character, 265
Gratuitous medical services, 82
Hallucinations, on muscular, by M. Semerie, 580
Indian army, sanitary state of, 651
Insane, the autobiography of, 333
——— colonization of, 576
Insanity, state of, in Scotland, 685
——————— Ireland, 685
——————— England, 685
——— American asylums for, 286
——— in France, 340
——— on the physiognomy of the insane, by Dr. Laurent, 495
——— "Spiritualism" as a cause of, 542
——— on a peculiar form of, caused by the political and social disturbances in France in 1848, by Dr. Bergeret, 608
"Lady Audley's Secret," 585
Laurent, Dr., on the physiology of the insane, 495
Libertinage, 728
Lectures, introductory, winter session, 1862, 161
Life, the mutual, of man, by F. Schnell, 286
Life, mental and physical, in relation to time, 224—459
Literary Notices:—
Gairdner's Clinical Medicine, 172
Brande and Taylor's Chemistry, 173
Condy's air and water: their impurities and purification, 177
Swedenborg, wisdom of, &c., by Medicus Cantabrigiensis, 177

Literary Notices :—
Harley, Dr., on jaundice, 563
English registrar-general's 24th annual report, 566
Scottish registrar-general's 4th annual report, 568
Laycock, Dr., on delirium tremens, 179
Year-book of medicine, 1862, 180
Hammond's military hygiene, 751
Smith, Dr. W. Abbotts, on human entozoa, 749
Barker (Dr.) on malaria and miasmata, 747
Waters (Dr.) on emphysema of the lungs, 748
Hogg, Mr. Jabez, on ophthalmoscopic surgery, 750
"Shirley Hall Asylum," 750
Fifth report of medical officer of privy council, 746
Fraser, Dr., on the Calabar bean, 749
Markham's travels in Peru and India while superintending the collection of cinchona plants, &c., 175
Mr. Curling on the rectum, 573
Dr. Hassall on the urine, 574
Roger on auscultation of the head, translated by Dr. Meadows, 749
Transactions of Epidemiological Society, pt. iii., vol. i., 570
Bray, Mr. Chas., on the philosophy of Necessity, 573
Lunacy, medical certificates in, 155
——— on the Stock Exchange, 329
——— state of, in Scotland, 534
——— state of, in Ireland, 685
——— state of, in England, 685
Mania, a rare case of, by Dr. H. G. Stewart, 254
——— Ephemera, by Dr. J. Crichton Browne, 45
M'Cormac, Dr. Henry, on the diet of the Insane, 219
M'Intosh, Dr. W. Carmichael, on morbid impulse, 101
Medical services, gratuitous, 82
Medicine, free-trade in, 36
Medico-legal trials, the Bridport murder, 376; the murder at Chatham, 383
Mental philosophy, Morell on inductive, 59
Mind, on the influence of material objects on, by Mr. J. Alexander Davies, 234
Morbid impulse, Dr. Carmichael M'Intosh on, 101, 305
Morell's inductive mental philosophy, 59
Moriz, Dr., on mental diseases of criminals, 758
Murchison on Continued Fevers, 702
Murders by lunatics, 742
Parigot, Dr., on Gheel, 190
Partial responsibility in lunacy and nervous disorders, by Dr. Legrand du Saulle, 668
Pharmacopœia, the new, 169
Physiognomy of insane, by Dr. Laurent, 495
Pregnant women, on the legal responsibility of, 694
Prison discipline and dietaries, 740
Prostitution, 736
Radcliffe, Mr., on Scutari, in March, 1863, 719
Rayer, M., on comparative medicine, 489
Richardson, Dr. B. W., on cooling of the body after death, 24
Royal Commission on Sanitary state of Indian army, 651
Sanitary instruction for the masses, 321
Schnell, Ferdinand, on the mutual life of man, 186
Scotland, the state of lunacy in, 534
Scutari, March, 1863, by J. N. Radcliffe, 719
Sedgwick, Mr., on the legal responsibility of pregnant women, 694
Semerie, M., on muscular hallucinations, 580
Sensation novels, 513
Session, winter, of 1862, opening of, 161
Small-pox, 562
Soul, on the unconscious life of, by Carus, 132
"Spiritualism" as a cause of insanity, 542
St. Thomas's Hospital, 169, 370, 558
Stewart, Dr. H. G., on a rare case of mania, 254
Stock Exchange, lunacy on, 329
Suicide in Bavaria, 579
——— baits for—"Lady Audley's Secret," and "Aurora Floyd," 585
——— in United States, 763
Surgery, the Dublin School of, 641
*Times* (the) on the lunacy of George III., 766
Trousseau on empiricism, 321
Typhus in Preston, 171
United States, suicide in, 763
Vaccination, state of public, 562
Ventilation of ships, 171
Vision, on a new theory of. By Mr. J. Alexander Davies, 92
Volition, on the nature of, psychologically and physiologically considered, by Mr. J. Lockhart Clarke, F.R.S., 1, 193
Volunteers, the Federal, sickness and mortality of, 123

END OF VOL. III.

